# Cell cycle and aging, morphogenesis, and response to stimuli genes are individualized biomarkers of glioblastoma progression and survival

**DOI:** 10.1186/1755-8794-4-49

**Published:** 2011-06-07

**Authors:** Nicola VL Serão, Kristin R Delfino, Bruce R Southey, Jonathan E Beever, Sandra L Rodriguez-Zas

**Affiliations:** 1 Department of Animal Sciences, University of Illinois at Urbana-Champaign, Urbana, Illinois, USA; 2 Department of Chemistry, University of Illinois at Urbana-Champaign, Urbana, Illinois, USA; 3 Department of Statistics, University of Illinois at Urbana-Champaign, Champaign, Illinois, USA; 4 Institute for Genomic Biology, University of Illinois at Urbana-Champaign, Urbana, Illinois, USA

## Abstract

**Background:**

Glioblastoma is a complex multifactorial disorder that has swift and devastating consequences. Few genes have been consistently identified as prognostic biomarkers of glioblastoma survival. The goal of this study was to identify general and clinical-dependent biomarker genes and biological processes of three complementary events: lifetime, overall and progression-free glioblastoma survival.

**Methods:**

A novel analytical strategy was developed to identify general associations between the biomarkers and glioblastoma, and associations that depend on cohort groups, such as race, gender, and therapy. Gene network inference, cross-validation and functional analyses further supported the identified biomarkers.

**Results:**

A total of 61, 47 and 60 gene expression profiles were significantly associated with lifetime, overall, and progression-free survival, respectively. The vast majority of these genes have been previously reported to be associated with glioblastoma (35, 24, and 35 genes, respectively) or with other cancers (10, 19, and 15 genes, respectively) and the rest (16, 4, and 10 genes, respectively) are novel associations. *Pik3r1*, *E2f3, Akr1c3*, *Csf1*, *Jag2*, *Plcg1*, *Rpl37a*, *Sod2*, *Topors*, *Hras*, *Mdm2, Camk2g*, *Fstl1*, *Il13ra1*, *Mtap *and *Tp53 *were associated with multiple survival events.

Most genes (from 90 to 96%) were associated with survival in a general or cohort-independent manner and thus the same trend is observed across all clinical levels studied. The most extreme associations between profiles and survival were observed for *Syne1*, *Pdcd4*, *Ighg1*, *Tgfa*, *Pla2g7*, and *Paics*. Several genes were found to have a cohort-dependent association with survival and these associations are the basis for individualized prognostic and gene-based therapies. *C2*, *Egfr*, *Prkcb*, *Igf2bp3*, and *Gdf10 *had gender-dependent associations; *Sox10*, *Rps20*, *Rab31*, and *Vav3 *had race-dependent associations; *Chi3l1*, *Prkcb*, *Polr2d*, and *Apool *had therapy-dependent associations. Biological processes associated glioblastoma survival included morphogenesis, cell cycle, aging, response to stimuli, and programmed cell death.

**Conclusions:**

Known biomarkers of glioblastoma survival were confirmed, and new general and clinical-dependent gene profiles were uncovered. The comparison of biomarkers across glioblastoma phases and functional analyses offered insights into the role of genes. These findings support the development of more accurate and personalized prognostic tools and gene-based therapies that improve the survival and quality of life of individuals afflicted by glioblastoma multiforme.

## Background

Glioblastoma multiforme (glioblastoma, World Health Organization grade IV astrocytoma) accounts for 15%-20% of all intracranial tumors and 50% of all brain malignancies [1]. This aggressive malignant type of primary brain tumor has swift and devastating consequences resulting in a median survival after diagnosis of one year [2,3,2]. Primary glioblastoma has a higher incidence in Caucasian men than in other racial and gender groups [4] although these differences may be confounded with differences in access to health care or diagnostic practices [5]. Also, the variation in response to glioblastoma therapies and similar median survival across therapies has prevented the identification of a therapy or therapies directly associated with glioblastoma survival [6-9].

Numerous studies have proposed biomarker genes that can be used to accurately predict the clinical course of glioblastoma [10-16]. Although some genes have been associated with the presence of glioblastoma, few have been identified as prognostic biomarkers of glioblastoma survival and fewer have been confirmed in independent reports. The limited reproducibility of gene-glioblastoma associations may be, in part, due to limited or no consideration of the clinical characteristics of the individuals studied, such as gender and therapy subject [17-19]. Another reason for the lack of confirmation of biomarker genes of glioblastoma may be the consideration of the association between glioblastoma and individual genes independently, although multiple genes acting in unison are known to influence this disease. Statistical reasons for this lack of confirmation include the analysis of gene expression levels in glioblastoma versus non-glioblastoma samples instead of analyzing survival, and the failure to correctly model the censored nature of the observations that may not exhibit the progression or death event by the end of the period considered. For example, The Cancer Genome Atlas Research Network (TCGA [20]) identified gene expression aberrations among the 206 glioblastoma cases considered but did not consider the age at glioblastoma death or progression, nor the clinical characteristics of the individuals studied.

The goal of this study was to identify general and clinical-dependent biomarker genes and biological processes of three complementary events: lifetime, overall and progression-free glioblastoma survival. A novel analytical strategy was developed to identify general and cohort-dependent associations between the biomarkers and the three glioblastoma events. Cross-validation and functional analysis further supported the identified biomarkers. The identification of gene biomarkers of glioblastoma survival supports the efficient follow-up studies using in vitro and in vivo experiments and augments the molecular toolbox that can be used to classify patients across and within cohort groups with respect to prognosis and the development of targeted treatments.

## Methods

### Data

Clinical and gene expression information from 320 individuals diagnosed with glioblastoma was obtained from the TCGA repository (September 2009 data freeze [21]. Protocols for specimen preparation and gene expression measurements are described in detail in the report by The Cancer Genome Atlas Research Network [20]. Briefly, a retrospective search in glioblastoma sample banks identified newly diagnosed glioblastoma cases based on surgical pathology reports and clinical records. Only samples that had demographic, clinical and pathological information, a minimum of 80% tumor nuclei, and a maximum of 50% necrosis, qualified for gene expression analyses.

The data included glioblastoma diagnostic and death records between the years 1989 and 2009. Clinical factors used to classify individuals into cohort groups were Gender (Male or Female), Race (White Caucasian or Other), Therapy received (R = radiation alone; CRnoT = chemo, radiation and not targeted therapy plus other therapy if present; CRT = chemo plus radiation and targeted therapy only; Other = any other combination of radiation, chemo, targeted, immune and hormonal therapy; or None = no therapy), and detection of glioblastoma progression or recurrence (ProgRec - Yes/No).

Three glioblastoma time-to-event variables were considered: lifetime survival (encompassing the period from birth to death), overall or post-diagnosis survival (encompassing the period from glioblastoma diagnosis to death) and post-diagnosis progression-free survival (encompassing the period from glioblastoma diagnosis to progression of glioblastoma or to recurrence of glioblastoma). The distribution of the observations among the levels of the clinical or cohort variables is presented in Table 1. There were 287 individuals with sufficient survival information for analysis. Three individuals were excluded from the progression-free survival analysis because of inconsistency in the dates for diagnosis and progression or recurrence.

**Table 1:** Median length of the hazard period and (relative) frequency of the individuals across clinical cohort levels

	Lifetime survival **(*n *= 287)**^**1**^		Overall survival **(*n *= 287)**^**2**^		Progression-free survival **(*n *= 284) **^**2**^	
Censored	(0.12)		(0.12)		(0.23)	
Lifetime	59.2		13.1		6.5	
Gender						
*Male*	58.8	(0.63)	13.6	(0.63)	6.6	(0.63)
*Female*	61.1	(0.37)	12.1	(0.37)	6.4	(0.37)
Race						
*White*	59.7	(0.77)	13.1	(0.77)	6.8	(0.77)
*Other*	57.7	(0.23)	12.6	(0.23)	5.3	(0.23)
Therapy						
*CRnoT*	57.7	(0.40)	15.7	(0.40)	8.0	(0.34)
*R*	60.7	(0.35)	12.3	(0.35)	5.3	(0.41)
*CRT*	53.4	(0.10)	15.4	(0.10)	6.8	(0.10)
*Other*	64.8	(0.08)	14.2	(0.08)	7.9	(0.08)
*None*	70.5	(0.07)	2.9	(0.07)	1.4	(0.07)
ProgRec						
*Yes*	57.6	(0.77)	15.1	(0.77)	-	(0.77)
*No*	64.8	(0.23)	5.9	(0.23)	-	(0.23)

^1 ^expressed in years;

^2 ^expressed in months.

Gene expression measurements were obtained using the Affymetrix HT HG-U133A platform, comprising 22,277 probe sets. The gene expression measurements were obtained in ten experimental batches, in which the percentage of individuals per batch ranged from 4.35% to 21.25%. For samples with multiple gene expression measurements, the correlation between measurements across microarrays was higher than 0.98 and, thus, the average expression was used to represent the sample. Raw expression data was log2 transformed and normalized using quantile normalization and GC-RMA [22] approaches implemented in Beehive [23].

In addition to detecting genes in the microarray platform associated with the glioblastoma survival, particular attention was given to genes known to be associated with glioblastoma and the association detected in this study. A list, including 123 genes known to be associated with glioblastoma were identified from the literature [20,24-27] and 51 genes in the KEGG glioma pathway [28], was compiled (see Additional file 1).

### Statistical Analysis

A five-step approach was used to reduce the dimensionality of the data set caused by the large number of probes and few records of the individuals in this experiment. First, a Cox proportional hazards survival analysis [29] was undertaken for each non-control probe in the microarray platform. The model included all the clinical variables with the profile of only one probe. This step allowed the selection of probes associated with each of the three survival variables at P-value < 0.01. This mild threshold was used to minimize the chances of false negative associations and evaluate in subsequent steps probes with strong or moderate associations with glioblastoma per se. The number of probes identified for lifetime survival, overall, and progression-free survival was 963, 839, and 1048 respectively. Second, for each one of the three glioblastoma time-to-event variables, the clinical variables and all remaining probes identified in the first step were included simultaneously in a Cox survival model. In this manner, the clinical variables were fixed component of the model and the probes associated with the survival variables were selected using a combination of forward and stepwise model selection methods. The forward selection method was used to add one probe at a time to the model containing the clinical variables using a significance level for entry of 30%. In the complementary stepwise selection method, the inclusion of probes followed the same rules as in the forward method but a probe only remained in the model if its P-value was lower than the significance levels for stay of 10%. Since these two selection methods could result in different models, a second stepwise selection was performed using the significant probes. This step allowed identifying broad or general associations between probe profiles and glioblastoma survival. Third, the interaction between the remaining probes and clinical variables was evaluated using the stepwise approach. This step permitted the detection of clinical or cohort-dependent associations between probe profiles and glioblastoma survival. The fourth stage of our approach aimed to select the significant probes from our list of 174 known genes associated with glioblastoma (see Additional file 1) fitting the probes and interaction with the clinical variables using the stepwise selection method. The consideration of the known probes alone aimed at minimizing the potential masking of associations by other probes in the model. Similarly to the previous step, in the final step the probes identified from both sets of analyses were combined and further streamlined using the stepwise method. This final step allowed the confirmation of prior probes associations reported in previous studies as well as the identification of novel associations. With respect to P-value threshold selection at each stage, a lenient first-stage threshold was used to capture most true positive associations at the expense of some false positives. The more stringent threshold used in the subsequent steps and repeated selection process minimized the number of false positives remaining in the index. Thus, this approach would have the same effect than reducing the threshold in the first step with the added benefit of minimizing the loss of true positives. Likewise, extending the first-stage threshold would have resulted in more false positives being considered in the second stage and higher risks of overparameterization.

In addition to a P-value, each probe had a hazard ratio (HR) estimate and associated 95% confidence interval limits. Hazard ratios below 1 indicate that the hazard under consideration decreases as the level of the gene increases. The proportional hazards assumption was assessed for the final predictive models corresponding to each survival variable based on the residuals. There was no evidence of departure from the assumptions for all the models reported. The association between survival and clinical and probe expression profiles was visualized by plotting the probability of survival predicted by the Cox model against time. For depiction purposes, individuals were divided into low and high probe expression groups that corresponded to the 25^th ^and 75^th ^percentile respectively given the median expression for all other probes in the predictive model. The survival curves were computed based on the information used to identify the significant gene associations. Biomarker genes resulting from the multi-stage approach were compared to previous reports of genes associated with glioblastoma or other cancers. The protein interaction resource at the NCBI Gene data base [30] was used to check that biomarkers not previously associated with cancer were also not indirectly associated with cancer through intermediate genes.

The genes identified by the five-step approach were compared to those resulting from a more conventional analysis using a one-step Cox survival analysis with a stringent cut-off (P-value < 0.0001).

### Functional and Gene Network Analyses

Identification of Gene Ontology (GO) categories (molecular function and biological process) and KEGG pathways represented among the significant genes associated with each glioblastoma survival variable was undertaken [31,32]. The representation of genes in the GO and KEGG pathway classes was evaluated using Fisher's exact (two-tailed) test and False Discovery Rate multiple test adjustment [33]. The relationships between the biomarker genes were further studied for the three glioblastoma survival variables and significant functional categories. The BisoGenet plug-in [34] from the Cytoscape software [35] was used to build and visualize the networks for each one of the three glioblastoma survival variables using the respective list of significant genes from the GO categories. All the available data sources in BisoGenet (including BIOGRID, DIP, BIND and others) were selected to generate the interactions. To facilitate the visualization of the networks, only interactions (edges) connecting two significant genes (nodes) directly or through an intermediate gene were depicted.

### Cross-validation

The associations between gene profiles and survival detected in this study were confirmed using a three-fold approach. First, a leave-one-out cross-validation (LOOCV) approach [36-38]. LOOCV is specially recommended in data sets of limited size, providing an almost unbiased estimator and identifying the same best classifiers as other X-fold training-test data partitions [38,39]. Validation of the predictive survival equation and biomarkers detected in a training data set on an independent test data set is desirable, followed by X-fold cross-validation on a particular data set. The representation of all cohort factors on both the training and test sets is necessary for unbiased evaluation of the biomarkers and to ensure that the detected biomarkers were not a spurious artifact of ignored cohort effects and for a fair evaluation of the training estimates. Consideration of race is particular critical for the validation of biomarkers detected in this study because lack of adjustment for this cohort factor could result in the identification of associations that are due to genetic background and not the particular gene expression profile.

For the X-fold validation approach, the specification of suitable training and testing data sets would have required at least 200 patients in each data set (5 individuals × 2 races × 2 genders × 5 therapies × 2 recurrence groups) and only 287individuals were available. The minimum of 5 individuals per group minimizes the risk of confounding between individual variation and cohort variation. Use of smaller data sets would have lead to low power and biased findings because of the ill-representation of individuals across cohort groups. Thus, the X-fold cross-validation could not be implemented. Likewise, the test of the predictive hazard equations (that include cohort factors) on an independent data set could not be implemented due to the lack of dataset with comparable cohort information or adequate structure that would minimize the risk of confounding between factors.

Accurate validation of associations between biomarkers and survival was attained using LOOCV discriminant analysis[40] that allows the assessment of the performance of biomarkers to classify individuals into high and low hazard (low and high survival, respectively). The same cohort information was used to obtain parameter estimates and to train the predictive hazard equations. For each survival variable, the median length of the period considered (age at death for lifetime survival; months from diagnosis to death for overall survival and; months from diagnosis to progression/recurrence for progression free survival) was calculated, and individuals were classified into either a high or low hazard group based on the median. The 20% of the individuals that had a length of period closest (higher or lower) to the median were not considered in order to minimize borderline cases that could affect the assessment of the model performance. Only non-censored records were used in the cross-validation analysis to favor unbiased classification. All individuals except for one were used to develop a new hazard index using the biomarkers previously detected and the new index was used to classify the remaining individuals. This leave-one-individual-out analysis was repeated for all individuals and the observed and predicted affiliations of the individuals to the high and low groups for each hazard were compared in order to assess the correct assignment rate.

Second, in addition to LOOCV, confirmation of the genes associated with the three glioblastoma hazards was investigated on the independent database REMBRANDT (REpository for Molecular BRAin Neoplasia DaTa) [41,42]. This database includes gene expression and survival information on 181 individuals diagnosed with glioblastoma. Third, a literature review was undertaken to identify independent studies that have reported associations between the genes associated with survival detected in this study and glioblastoma or other cancer types.

## Results

### Confirmed and Novel Biomarkers of Glioblastoma

The median length of the periods associated with lifetime, overall, and progression-free survival across and within clinical or cohort group are presented in Table 1. The age of the individuals at death or at the end of the considered period ranged from 14 to 87 years with a median age of 60 years. The median survival length was 59 years, 13 months and 7 months for lifetime, overall, and progression-free survival, respectively.

A total of 168 significant associations between expression profiles and glioblastoma survival (61, 47 and 60 associations for lifetime, overall, and progression-free survival, respectively) from 139 genes were identified. Among these, 10 associations are borderline significant (0.1 < P-value < 0.05) and are included in the tables in support of other more significant associations.

The vast majority of the genes associated with glioblastoma survival have been previously reported to be associated with glioblastoma (35, 24, and 35 genes, respectively) or with another cancer (10, 19, and 15 genes, respectively) and the rest (16, 4, and 10 genes, respectively) exhibited novel associations with glioblastoma. Table 2 presents the distribution of genes and probes associated with more than one hazard. Cohort-independent and cohort-dependent associations, respectively, were uncovered for lifetime (Tables 3 and 4), overall (Tables 5 and 6), and progression-free (Tables 7 and 8) survival. Cohort-independent associations represented 90%, 96% and 92% of the significant gene associations for lifetime, overall, and progression-free survival, respectively.

**Table 2:** Genes and probes represented more than one time within or across the glioblastoma survival events

Gene	Lifetime Survival	Overall Survival	Progression-free survival
*Actr2*		200727_s_at	
		200729_s_at	
*Akr1c3*	209160_at	209160_at	
*App*			211277_x_at
			214953_s_at
*Camk2b*			211483_x_at
			209956_s_at
*Camk2g*	212757_s_at		214322_at
	214322_at		
*Cdc42*	208727_s_at		
	208728_s_at		
	214230_at		
*Chi3l1*	216546_s_at		
	209396_s_at		
*Csf1*	207082_at	209716_at	
*E2f3*	203692_s_at	203693_s_at	203693_s_at
*Egfr*	211551_at		
	211607_x_at		
*Fstl1*	208782_at		208782_at
*Hras*		212983_at	212983_at
*Ighg1*		211908_x_at	
		211693_at	
*Il13ra1*	210904_s_at		211612_s_at
*Jag2*	32137_at	209784_s_at	
*Mdm2*		217373_x_at	217373_x_at
*Mtap*	204956_at		204956_at
*Pik3r1*	212240_s_at	212249_at	212239_at
*Plcg1*	216551_x_at	216551_x_at	
*Prkcb*	207957_s_at		
	209685_s_at		
*Rpl37a*	213459_at	213459_at	
*Sod2*	221477_s_at	215078_at	
*Timp3*			201148_s_at
			201150_s_at
*Topors*	204071_s_at	204071_s_at	
*Tp53*	211300_s_at		211300_s_at

**Table 3:** Genes that have a general association (P-value < 0.05) with the lifetime glioblastoma survival

Gene Symbol	Probe Identifier	P-value	**Hazard Ratio**^**1**^	Relevant literature references
*Syne1*	209447_at	<.0001	0.17 (0.10-0.32)	[60]^O^
*E2f3*	203692_s_at	<.0001	0.26 (0.15-0.44)	[28]^G^
*Fstl1*	208782_at	<.0001	0.31 (0.22-0.42)	[25]^G^
*Ep300*	213579_s_at	<.0001	0.34 (0.29-0.57)	[26]^G^
*Gigyf2*	212261_at	<.0001	0.39 (0.26-0.58)	n/a
*Topors*	204071_s_at	<.0001	0.41 (0.29-0.59)	[26]^G^
*Chst4*	220446_s_at	0.0989^2^	0.44 (0.17-1.16)	[89]^O^
*Sar1a*	201543_s_at	<.0001	0.44 (0.29-0.66)	n/a
*Il13ra1*	210904_s_at	<.0001	0.47 (0.36-0.60)	[24]^G^
*Sod2*	221477_s_at	<.0001	0.47 (0.37-0.59)	[25]^G^
*Rab15*	221810_at	<.0001	0.48 (0.34-0.69)	n/a
*Timm23*	218118_s_at	0.0239	0.50 (0.27-0.91)	n/a
*Kcnj4*	208359_s_at	<.0001	0.50 (0.38-0.66)	n/a
*Rpl37a*	213459_at	0.0023	0.51 (0.33-0.79)	[90]^G^
*Camk2g*	214322_at	0.0135	0.53 (0.32-0.88)	[56]^G^
*Plcg1*	216551_x_at	0.0068	0.55 (0.35-0.85)	[26]^G^
*Slc43a3*	213113_s_at	0.0004	0.56 (0.40-0.77)	n/a
*Cdc42*	208727_s_at	<.0001	0.57 (0.45-0.73)	[26]^G^
*Csf1*	207082_at	0.0092	0.58 (0.38-0.88)	[26]^G^
*Ccnb2*	202705_at	0.0118	0.60 (0.40-0.89)	[91]^G^
*Tlk2*	212997_s_at	0.0004	0.64 (0.49-0.82)	n/a
*Mtap*	204956_at	0.0091	0.67 (0.49-0.91)	[26]^G^
*Egfr*	211551_at	0.0743^2^	0.68 (0.45-1.04)	[24]^G^
*Akt2*	211453_s_at	0.0292	0.68 (0.48-0.96)	[86]^G^
*Akr1c3*	209160_at	<.0001	0.70 (0.62-0.81)	[26]^G^
*Tp53*	211300_s_at	0.0215	0.76 (0.60-0.96)	[25]^G^
*Igf1*	209541_at	0.0183	0.76 (0.61-0.95)	[26]^G^
*Rpl10*	221989_at	0.0392	0.80 (0.64-0.99)	[24]^G^
*Arhgef4*	205109_s_at	0.0647^2^	0.80 (0.64-1.01)	n/a
*Cdc42*	214230_at	0.0554^2^	0.82 (0.67-1.00)	[26]^G^
*Chi3l1*	216546_s_at	0.061	0.87 (0.75-1.00)	[70]^G^
*Ppbp*	214146_s_at	0.012	1.16 (1.03-1.30)	n/a
*Cdkn2a*	209644_x_at	0.0003	1.18 (1.08-1.29)	[92]^G^
*Wdr67*	214061_at	0.0237	1.30 (1.03-1.63)	[93]^O^
*Tspyl5*	213122_at	0.0003	1.34 (1.14-1.56)	n/a
*Usf2*	215737_x_at	<.0001	1.42 (1.19-1.69)	[94]^O^
*Camk2g*	212757_s_at	0.0078	1.54 (1.12-2.13)	[56]^G^
*Pik3r1*	212240_s_at	0.0022	1.67 (1.20-2.32)	[20]^G^
*Akt1*	207163_s_at	0.0005	1.70 (1.26-2.30)	[84]^O^
*Rac2*	213603_s_at	0.0001	1.74 (1.31-2.31)	[95]^G^
*Six6*	207250_at	<.0001	1.82 (1.45-2.28)	[96]^O^
*Spg21*	217827_s_at	0.0387	1.91 (1.03-3.52)	n/a
*Wdyhv1*	219060_at	0.0015	1.95 (1.29-2.94)	n/a
*Uros*	203031_s_at	0.0067	2.37 (1.27-4.42)	n/a
*Lin7c*	219399_at	0.0002	2.40 (1.51-3.80)	[97]^O^
*Ros1*	207569_at	<.0001	2.58 (1.73-3.85)	[98]^O^
*Cdk2*	204252_at	<.0001	2.74 (1.78-4.21)	[91]^G^
*Jag2*	32137_at	<.0001	2.78 (1.86-4.14)	[24]^G^
*Kiaa0090*	212395_s_at	<.0001	2.89 (1.87-4.47)	n/a
*Ccnb1*	214710_s_at	<.0001	3.16 (2.00-4.98)	[91]^G^
*Scn5a*	207413_s_at	<.0001	3.21 (1.79-5.74)	n/a
*Col14a1*	212865_s_at	<.0001	3.30 (1.93-5.63)	[99]^O^
*Hoxa10*	213147_at	<.0001	3.30 (1.93-5.65)	[100]^O^
*Cdc42*	208728_s_at	<.0001	3.94 (2.12-7.32)	[26]^G^
*Pdcd4*	202731_at	<.0001	4.68 (3.01-7.28)	[26]^G^

n/a, No association with any type of cancer found in literature;

^1 ^Hazard ratio estimate (95% confidence interval);

^2 ^Borderline significant (P-value < 0.1) included for completeness;

^G ^Gene confirmed in an independent glioblastoma multiforme study; the number between square brackets denotes the corresponding literature reference;

^O ^Gene confirmed in an independent study on any other type of cancer; the number between square brackets denotes the corresponding literature reference.

**Table 4:** Genes that have a cohort-dependent association (P-value < 0.05) with the lifetime glioblastoma survival

Gene Symbol	Probe Identifier	Clinical Cohort	P-value	Level of Clinical Cohort	**Hazard Ratio**^**1**^	Relevant literature references
*Prkcb*^*2*^	207957_s_at	Gender	<.0001	Male	0.36 (0.24-0.55)	[28]^G^
				Female	1.27 (0.84-1.93)	
		Therapy	0.0006	None	0.38 (0.25-0.60)	
				CRnoT	0.51 (0.36-0.73)	
				R	0.64 (0.46-0.88)	
				CRT	0.71 (0.44-1.13)	
				Other	0.75 (0.43-1.32)	
*Sox10*	209843_s_at	Race	0.0018	White	0.55 (0.44-0.68)	[10]^G^
				Other	1.08 (0.72-1.62)	
*Egfr*	211607_x_at	Gender	<.0001	Male	0.60 (0.50-0.72)	[24]^G^
				Female	0.88 (0.74-1.04)	
*Chi3l1*	209396_s_at	Therapy	0.0006	CRT	1.27 (0.96-1.70)	[70]^G^
				R	1.28 (1.07-1.52)	
				Other	1.31 (1.04-1.66)	
				CRnoT	1.53 (1.31-1.79)	
				None	2.42 (1.56-3.75)	
*C2*	203052_at	Gender	0.0033	Female	1.30 (1.03-1.65)	n/a
				Male	1.93 (1.56-2.39)	
*Prkcb*	209685_s_at	Gender	<.0001	Female	1.31 (0.79-2.14)	[28]^G^
				Male	5.21 (3.16-8.61)	

n/a, No association with any type of cancer found in literature;

^1 ^Hazard ratio estimate (95% confidence interval);

^2 ^Interaction with a single clinical cohort factor;

^G ^Gene confirmed in an independent glioblastoma multiforme study; the number between square brackets denotes the corresponding literature reference;

^O ^Gene confirmed in an independent study on any other type of cancer; the number between square brackets denotes the corresponding literature reference.

**Table 5:** Genes that have a general association (P-value < 0.05) with the overall glioblastoma survival

Gene Symbol	Probe Identifier	P-value	**Hazard Ratio**^**1**^	Relevant literature references
*Tgfa*	205015_s_at	0.0002	0.12 (0.04-0.37)	[28]^G^
*Sirpa*	202895_s_at	<.0001	0.24 (0.14-0.41)	[26]^G^
*Ctbp2*	210835_s_at	<.0001	0.28 (0.16-0.48)	[101]^O^
*Eef1e1*	213907_at	<.0001	0.37 (0.23-0.61)	[102]^O^
*Mapk3*	212046_x_at	0.0041	0.43 (0.24-0.76)	[103]^O^
*Actr2*	200727_s_at	<.0001	0.43 (0.29-0.63)	[24]^G^
*Igh@*	211637_x_at	0.0167	0.44 (0.23-0.86)	n/a
*Plcg1*	216551_x_at	<.0001	0.46 (0.31-0.68)	[26]^G^
*Mgat3*	209764_at	<.0001	0.51 (0.37-0.71)	[26]^G^
*Lrp10*	201412_at	0.0041	0.60 (0.42-0.85)	[24]^G^
*Idh1*	201193_at	0.0051	0.60 (0.42-0.86)	[24]^G^
*Tmem8b*	207839_s_at	<.0001	0.60 (0.46-0.77)	n/a
*Ccna2*	203418_at	<.0001	0.60 (0.49-0.75)	[104]^O^
*Topors*	204071_s_at	0.0007	0.61 (0.46-0.81)	[26]^G^
*Rpl37a*	213459_at	0.0164	0.66 (0.47-0.93)	[90]^G^
*Mdm2*	217373_x_at	<.0001	0.69 (0.61-0.78)	[26]^G^
*E2f3*	203693_s_at	0.0672^2^	0.75 (0.55-1.02)	[28]^G^
*Mdfic*	211675_s_at	0.0006	0.78 (0.68-0.90)	[105]^O^
*Sod2*	215078_at	<.0001	0.80 (0.73-0.88)	[25]^G^
*Akr1c3*	209160_at	0.0014	0.83 (0.73-0.93)	[26]^G^
*Thbs4*	204776_at	0.0007	1.18 (1.07-1.30)	[106]^O^
*Shc3*	206330_s_at	0.0031	1.32 (1.10-1.59)	[28]^G^
*Pik3r1*	212249_at	0.0145	1.34 (1.06-1.69)	[20]^G^
*Nkx2-5*	206578_at	0.0027	1.38 (1.12-1.70)	[107]^O^
*Hras*	212983_at	0.0187	1.42 (1.06-1.90)	[85]^G^
*Bhlhb9*	213709_at	0.0192	1.42 (1.06-1.92)	[108]^O^
*C9orf95*	219147_s_at	0.0004	1.43 (1.17-1.73)	[109]^O^
*C17orf101*	219254_at	0.0085	1.46 (1.10-1.94)	[110]^O^
*Nol3*	59625_at	<.0001	1.46 (1.21-1.76)	[111]^O^
*Rangap1*	212125_at	0.0225	1.47 (1.06-2.05)	[26]^G^
*Ftsj2*	222130_s_at	0.017	1.48 (1.07-2.05)	[112]^O^
*Rrm1*	201476_s_at	0.0006	1.49 (1.19-1.87)	[27]^G^
*Jag2*	209784_s_at	0.0351	1.63 (1.03-2.57)	[24]^G^
*Tnpo1*	212635_at	0.0054	1.89 (1.20-2.96)	n/a
*Myo7a*	211103_at	0.0033	1.97 (1.25-3.10)	[113]^O^
*Actr2*	200729_s_at	0.0001	2.18 (1.47-3.23)	[24]^G^
*Csf1*	209716_at	<.0001	2.33 (1.65-3.27)	[26]^G^
*Ank1*	208352_x_at	0.0003	2.38 (1.49-3.82)	[24]^G^
*B3galnt1*	211379_x_at	<.0001	2.40 (1.76-3.28)	[114]^O^
*Kras*	214352_s_at	0.002	2.44 (1.38-4.31)	[85]^G^
*Ewsr1*	210012_s_at	0.0005	2.49 (1.49-4.15)	[26]^G^
*Sec24c*	202361_at	<.0001	2.84 (1.76-4.60)	n/a
*Rpl10l*	217559_at	<.0001	2.95 (1.83-4.74)	[115]^O^
*Ighg1*	211908_x_at	0.0007	3.41 (1.68-6.93)	[116]^O^
*Ighg1*	211693_at	0.0007	4.33 (1.86-10.04)	[116]^O^

n/a, No association with any type of cancer found in literature;

^1 ^Hazard ratio estimate (95% confidence interval);

^2 ^Borderline significant (P-value < 0.1) included for completeness;

^G ^Gene confirmed in an independent glioblastoma multiforme study; the number between square brackets denotes the corresponding literature reference;

^O ^Gene confirmed in an independent study on any other type of cancer; the number between square brackets denotes the corresponding literature reference.

**Table 6:** Genes that have a cohort-dependent association (P-value < 0.05) with the overall glioblastoma survival

Gene Symbol	Probe Identifier	Clinical Cohort	P-value	Level of Clinical Cohort	**Hazard Ratio**^**1**^	Relevant literature references
*Polr2d*	214144_at	Therapy	0.0044	Other	0.35 (0.18-0.70)	[117]^O^
				R	0.50 (0.31-0.81)	
				CRT	0.68 (0.38-1.21)	
				None	0.77 (0.43-1.39)	
				CRnoT	0.93 (0.58-1.5)	
*Igf2bp3*	203820_s_at	Gender	0.0146	Female	1.02 (0.84-1.24)	[118]^O^
				Male	1.29 (1.12-1.49)	

n/a, No association with any type of cancer found in literature;

^1 ^Hazard ratio estimate (95% confidence interval);

^G ^Gene confirmed in an independent glioblastoma multiforme study; the number between square brackets denotes the corresponding literature reference;

^O ^Gene confirmed in an independent study on any other type of cancer; the number between square brackets denotes the corresponding literature reference.

**Table 7:** Genes that have a general association (P-value < 0.05) with the progression-free glioblastoma survival

Gene Symbol	Probe Identifier	P-value	**Hazard Ratio**^**1**^	Relevant literature references
*Pla2g7*	206214_at	<.0001	0.11 (0.05-0.23)	[119]^O^
*Pdgfb*	216061_x_at	<.0001	0.18 (0.09-0.35)	[28]^G^
*Calm2*	207243_s_at	0.0011	0.22 (0.09-0.54)	[28]^G^
*Timp3*	201148_s_at	<.0001	0.26 (0.16-0.41)	[26]^G^
*Agpat1*	215535_s_at	<.0001	0.27 (0.15-0.51)	n/a
*Ifngr1*	202727_s_at	<.0001	0.32 (0.19-0.53)	[27]^G^
*Pvr*	214444_s_at	<.0001	0.32 (0.19-0.54)	[26]^G^
*Ndufv1*	208714_at	0.0002	0.33 (0.19-0.59)	n/a
*Fgfr2*	211401_s_at	<.0001	0.39 (0.26-0.57)	[26]^G^
*E2f3*	203693_s_at	<.0001	0.42 (0.29-0.60)	[28]^G^
*Pold2*	201115_at	0.0007	0.43 (0.27-0.70)	[26]^G^
*Calm3*	200622_x_at	0.0001	0.43 (0.28-0.67)	[28]^G^
*Tp53*	211300_s_at	<.0001	0.43 (0.29-0.64)	[25]^G^
*Raf1*	201244_s_at	0.0141	0.46 (0.25-0.85)	[26]^G^
*Pknox2*	219046_s_at	<.0001	0.46 (0.33-0.62)	[120]^O^
*App*	214953_s_at	0.0016	0.47 (0.30-0.75)	[24]^G^
*Fstl1*	208782_at	<.0001	0.47 (0.35-0.62)	[25]^G^
*Camk2b*	211483_x_at	0.0008	0.54 (0.38-0.77)	[28]^G^
*Pten*	204053_x_at	0.0003	0.60 (0.46-0.79)	[26]^G^
*Mdm2*	217373_x_at	<.0001	0.66 (0.57-0.76)	[26]^G^
*Ccnd1*	208711_s_at	0.0273	0.80 (0.66-0.98)	[28]^G^
*Hspa1a*/*Hspa1b*	202581_at	0.0529	0.82 (0.67-1. 00)	[24]^G^
*Cd24*	208650_s_at	0.0069	0.85 (0.75-0.95)	[121]^O^
*Clec2b*	209732_at	0.0645^2^	1.19 (0.99-1.44)	[122]^O^
*Cav2*	203324_s_at	0.0024	1.25 (1.08-1.44)	[123]^O^
*Snx10*	218404_at	<.0001	1.34 (1.16-1.57)	[124]^O^
*Wee1*	215711_s_at	0.0083	1.37 (1.08-1.74)	[27]^G^
*Hras*	212983_at	0.0802^2^	1.49 (0.95-2.33)	[85]^G^
*Mns1*	219703_at	0.0053	1.51 (1.13-2.02)	n/a
*Ppp1r15a*	37028_at	0.011	1.54 (1.10-2.15)	n/a
*App*	211277_x_at	0.0811^2^	1.56 (0.95-2.56)	[24]^G^
*Fadd*	202535_at	0.0934^2^	1.57 (0.93-2.65)	[125]^O^
*Pik3r1*	212239_at	0.018	1.60 (1.08-2.36)	[20]^G^
*Mmp14*	217279_x_at	0.0182	1.66 (1.09-2.52)	[24]^G^
*Mtap*	204956_at	0.0016	1.66 (1.21-2.27)	[26]^G^
*Il13ra1*	211612_s_at	0.0003	1.72 (1.28-2.32)	[24]^G^
*Kcnj13*	210179_at	0.0235	1.74 (1.08-2.82)	[126]^O^
*Clip3*	212358_at	0.0022	1.75 (1.22-2.50)	n/a
*Aanat*	207225_at	0.0114	1.78 (1.14-2.79)	[127]^O^
*Camk2g*	214322_at	0.0024	1.86 (1.24-2.78)	[56]^G^
*Prkca*	215195_at	0.0005	1.90 (1.32-2.73)	[28]^G^
*Kdm6b*	41386_i_at	0.0003	2.03 (1.39-2.96)	n/a
*Zfy*	207246_at	0.0016	2.06 (1.31-3.22)	[128]^O^
*Smarcb1*	212167_s_at	0.0004	2.06 (1.38-3.07)	[26]^G^
*Utp20*	209725_at	<.0001	2.08 (1.46-2.98)	n/a
*Igl@*	211655_at	0.0209	2.22 (1.13-4.38)	[129]^O^
*Atf5*	204998_s_at	<.0001	2.31 (1.72-3.11)	[130]^G^
*Shox*	207570_at	<.0001	2.66 (1.73-4.07)	[24]^G^
*Loc283079*	215929_at	0.0071	2.73 (1.31-5.69)	n/a
*Ung*	202330_s_at	0.0001	2.79 (1.66-4.68)	[27]^G^
*Hnrnpd*	213359_at	<.0001	2.94 (1.91-4.52)	n/a
*Camk2b*	209956_s_at	<.0001	3.02 (2.13-4.29)	[28]^G^
*Timp3*	201150_s_at	<.0001	3.10 (1.88-5.11)	[26]^G^
*Nras*	202647_s_at	<.0001	3.93 (2.60-5.95)	[28]^G^
*Paics*	214664_at	<.0001	5.28 (3.13-8.91)	[66]^O^

n/a, No association with any type of cancer found in literature;

^1 ^Hazard ratio estimate (95% confidence interval);

^2 ^Borderline significant (P-value < 0.1) included for completeness;

^G ^Gene confirmed in an independent glioblastoma multiforme study; the number between square brackets denotes the corresponding literature reference;

^O ^Gene confirmed in an independent study on any other type of cancer; the number between square brackets denotes the corresponding literature reference.

**Table 8:** Genes that have a cohort-dependent association (P-value < 0.05) with progression-free glioblastoma survival

Gene Symbol	Probe Identifier	Clinical Cohort	P-value	Level of Clinical Cohort	**Hazard Ratio**^**1**^	Relevant literature references
*Gdf10*	206159_at	Gender	0.0317	Male	0.37 (0.23-0.60)	n/a
				Female	0.80 (0.45-1.42)	
*Vav3*	218807_at	Race	0.008	Other	0.41 (0.29-0.59)	[81]^G^
				White	0.68 (0.55-0.85)	
*Rps20*	216246_at	Race	0.0003	Other	0.75 (0.39-1.44)	[75]^O^
				White	1.83 (1.03-3.24)	
*Rab31*	217764_s_at	Race	<.0001	White	1.47 (0.93-2.30)	[74]^O^
				Other	7.72 (3.71-16.07)	
*Apool*	213289_at	Therapy	0.0026	R	1.64 (1.13-2.38)	[131]^O^
				None	1.93 (0.63-5.98)	
				CRnoT	2.23 (1.55-3.20)	
				Other	3.86 (1.83-8.13)	
				CRT	4.82 (2.69-8.63)	

n/a, No association with any type of cancer found in literature;

^1 ^Hazard ratio estimate (95% confidence interval);

^G ^Gene confirmed in an independent glioblastoma multiforme study; the number between square brackets denotes the corresponding literature reference

^O ^Gene confirmed in an independent study on any other type of cancer; the number between square brackets denotes the corresponding literature reference.

The five-step approach was consistently superior to a one-step Cox analysis with more stringent P-value < 0.001 on all three survival indicators. For the three variables studied; lifetime, overall, and progression-free survival, the simpler approach identified 60, 71 and 67 probes of which 19, 17, and 23 respectively overlapped with the corresponding 61, 47, and 60 probes identified in the five-step analyses of the three survival indicators. Of the 139 probes identified by the simpler approach and not identified by our approach, the vast majority (123 probes across all three variables) have not been associated with glioblastoma and could not be confirmed.

### Genes Associated with Lifetime Death Hazard

**Figure 1:**
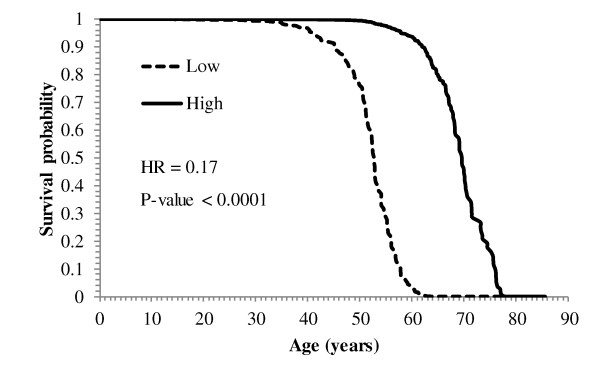
**Probability of lifetime glioblastoma survival across age for spectrin repeat containing, nuclear envelope 1 (*Syne1*)**. Probability of glioblastoma survival across age for individuals with Low (25^th ^percentile) and High (75^th ^percentile) expression level of spectrin repeat containing, nuclear envelope 1 (*Syne1*). With a lower hazard estimate (HR = 0.17), the probability of survival of individuals with high levels of *Syne1 *remains higher across age.

Sixty-one gene profiles, representing 55 genes, were associated with lifetime survival. An increase in the level of expression of 31 genes was associated with a decrease in HR, with estimates ranging from 0.17 (*Syne1*) to 0.87 (*Chi3l1*). The changes in survival across levels of gene expression and clinical variables for the population under consideration were visualized using survival plots. The decline on the probability of lifetime survival across age (in years) for individuals with high (75^th ^percentile) and low (25^th ^percentile) levels of *Syne1 *is depicted in Figure 1. Consistent with the hazard ratio estimate (HR = 0.17, P-value < 0.0001), the probability of survival of individuals with high levels of *Syne1 *remains higher across age. Individuals with high and low levels of *Syne1 *have a survival probability of 50% at 69 and 52 years of age, respectively. The opposite trend was observed in the remaining 24 profiles that have hazard ratio estimates ranging from 1.16 (*Ppbp*) to 4.7 (*Pdcd4*).

**Figure 2:**
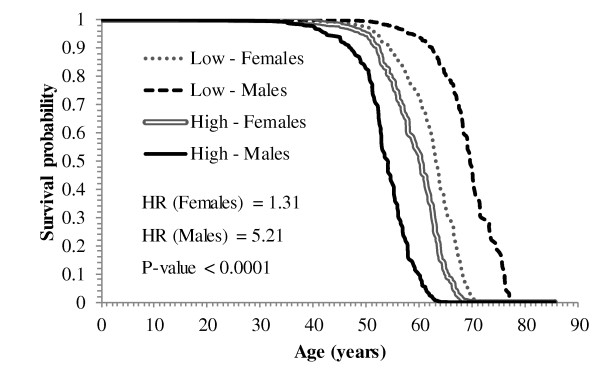
**Probability of lifetime glioblastoma survival across age in females and males for protein kinase, C beta (*Prkcb*)**. Probability of glioblastoma survival across age for Females and Males with Low (25^th ^percentile) and High (75^th ^percentile) expression level of protein kinase, C beta (*Prkcb*). Consistent with the hazard ratio estimates for females (HR = 1.31) and males (HR = 5.21), the probability of survival in individuals with high levels of *Prkcb *declines before than in individuals with lower levels of *Prkcb*. Due to the significant interaction between the expression of *Prkcb *and gender, the probability of survival for females with high level of the gene declines faster than the probability of survival for males with low level of the gene.

Among the genes exhibiting cohort-dependent associations with lifetime survival (Table 4), the drop in the probability of lifetime survival across age (in years) for females and males with high (75^th ^percentile) and low (25^th ^percentile) levels of *Prkcb *209685_s_at is portrayed in Figure 2. Consistent with the hazard ratio estimates for females (HR = 1.31) and males (HR = 5.21), the probability of survival declines faster in males with high levels of *Prkcb *than females with low levels of this gene.

### Genes Associated with Overall Survival

A total of 45 genes were associated with overall survival (Tables 5 and 6). Among the cohort-independent associations, an increase in the levels of 20 genes was associated with a decrease in overall hazard with HR ranging from 0.12 (*Tgfa*) to 0.83 (*Akr1c3*). On the other hand, an increase in the level of 25 genes was associated with an increase in overall hazard with HR ranging from 1.18 (*Thbs4*) to 4.33 (*Ighg1*). Among the cohort-dependent associations, the hazard increased more in males (HR = 1.29) than in females (HR = 1.02) per unit increase in the levels of *Igf2bp3*.

### Genes Associated with Progression-free Survival

**Figure 3:**
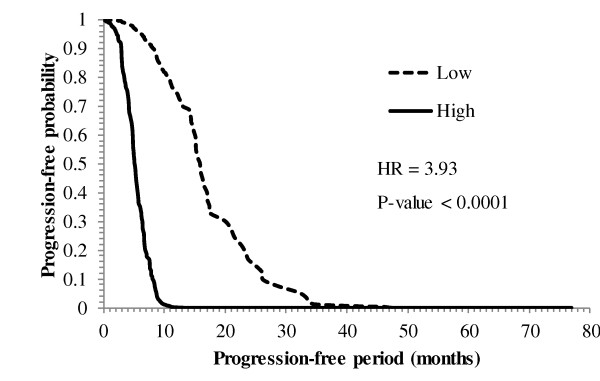
**Progression-free probability across post-diagnosis months for neuroblastoma RAS viral (v-RAS) oncogene homolog (*Nras*)**. Progression-free probability across post-diagnosis months for individuals with Low (25^th ^percentile) and High (75^th ^percentile) expression level of neuroblastoma RAS viral (v-RAS) oncogene homolog (*Nras*). With a high hazard estimate (HR = 3.93), the progression-free probability falls faster in individuals with high expression levels of *Nras*.

Of the 60 probes (corresponding to 57 genes) associated with progression-free survival, 55 had general associations and 5 had cohort-dependent associations (Tables 7 and 8). Among the genes that have cohort-independent associations, an increase in the level of 23 genes was associated with a decrease in HR, ranging from 0.11 (*Pla2g7*) to 0.85 (*Cd24*). For the remaining 32 genes, an increase in the level of expression was associated with an increase in the progression-free HR ranging from 1.19 (*Clec2b*) to 5.28 (*Paics*). The decline in the progression-free survival probability across time (in months) for individuals with high (75^th ^percentile) and low (25^th ^percentile) levels of neuroblastoma RAS viral (v-RAS) oncogene homolog (*Nras*) is depicted in Figure 3. Consistent with the hazard ratio estimate (HR = 3.93, P-value < 0.0001), the progression-free survival probability falls faster in individuals with high expression levels of *Nras*. With regard to the cohort-dependent association with progression-free survival, an increase in the expression of *Gdf10 *was associated with a higher decrease of the hazard ratio in males (HR = 0.37) than in females (HR = 0.80).

### Gene That Have Multiple Probes and Hazard Ratios

When multiple probes of the same gene had opposite associations with the glioblastoma hazard (e.g. HR > 1 for probe 1 and HR < 1 for probe 2), the disagreements were resolved by assessing the dependability of each probe. Information on dissenting probes is briefly summarized here. Probe 214322_at, of *Cam2kg*, was obtained from an ovary EST and thus is less reliable in respect to brain cancer than probe 212757_s_at. Probe 208728_s_at, of *Cdc42*, is expected to be more reliable than 208727_s_at because the former was obtained from an mRNA sequence that has double the length than the later. Probe 200729_s_at, of *Actr2*, corresponds to an mRNA and is more reliable than 200727_s_at, which corresponds to an EST from mixed tissues. Probe 210904_s_at, of *Il13ra1*, corresponds to a cluster of mRNA assigned to this gene in the NCBI-Gene database; meanwhile, probe 211612_s_at pertains to a single mRNA not assigned to the gene and is considered less reliable. Other probes include 201148_s_at of *Timp3*, which corresponds to a pancreatic EST, 200727_s_at of *Actr2*, which corresponds to a mixed tissue EST, and 209956_s_at of *Camk2b*, which corresponds to a proline rich sequence.

### Functional and Gene Network Analyses

**Figure 4:**
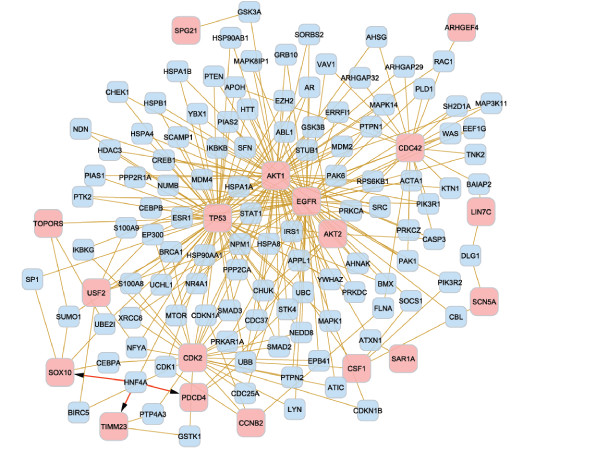
**Gene network from the functional analysis of lifetime glioblastoma survival**. Interaction between the significant genes from the functional analysis of lifetime glioblastoma death. The gold edges represent protein interactions whereas the red edges represent interaction of the HNF4A protein with the DNA of the genes *Pdcd4*, *Sox10 *and *Timm23*. Of the 24 genes from Table 9, 18 (pink nodes) interact among each other in a direct way or through an intermediate gene (blue nodes).

**Figure 5:**
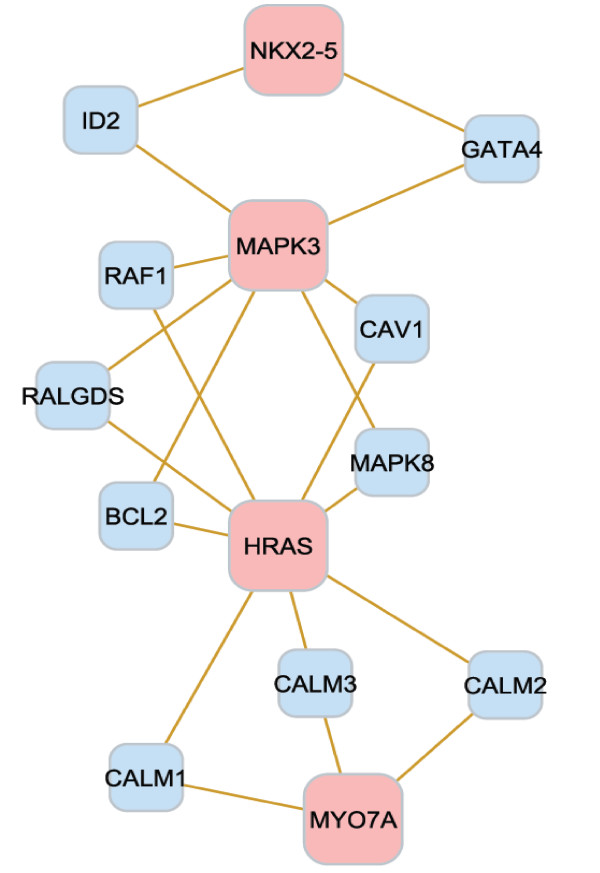
**Gene network from the functional analysis of overall glioblastoma survival**. Relationship between the significant genes from the functional analysis of overall survival. Of the nine genes from Table 10, four (pink nodes) interact among each other in a direct way or through an intermediate gene (blue nodes).

**Figure 6:**
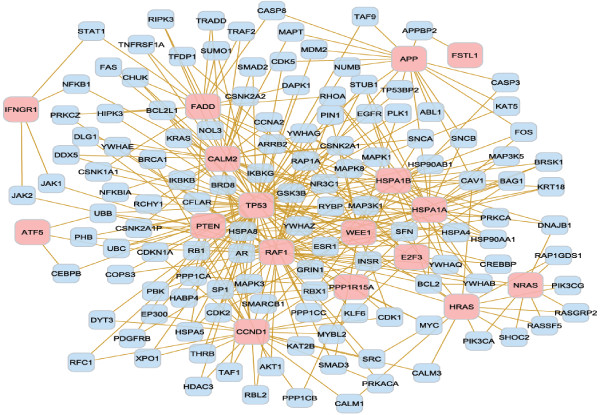
**Gene network from the functional analysis of progression-free survival**. Relationship between the significant genes from the functional analysis of progression-free survival. Of the 19 genes from Table 11, 17 (pink nodes) interact among each other in a direct way or through an intermediate gene (blue nodes).

The GO categories enriched (FDR adjusted P-value < 0.1, ≥ 3 genes/category) among the genes associated with each of the three glioblastoma survival variables are summarized in Tables 9, 10, and 11. The functional analysis revealed nine, two and ten biological processes enriched among the genes associated with lifetime, overall, and progression-free survival respectively, and three molecular functions enriched among the genes associated with progression-free survival. The biological processes of cell cycle (GO:0007049) and death (GO:0016265) were over-represented among the genes associated with the lifetime and progression-free survivals. The gene networks for the significant genes from the functional analyses associated with lifetime, overall, and progression-free survival are depicted in Figures 4, 5 and 6, respectively.

**Table 9:** Gene Ontology categories enriched among the genes associated with lifetime glioblastoma survival^1^

Gene Ontology	Level	Term	P-value	FDR adjusted P-value	Number of genes	Genes
Biological process	3	aging (GO:0007568)	2.13E-05	1.62E-03	3	*Pdcd4*, *Cdkn2a*, *Tp53*
		regulation of biological process (GO:0050789)	2.77E-04	1.50E-02	20	*Usf2*, *Cdkn2a*, *Ccnb2*, *Akt2*, *Tp53*, *Cdc42*, *Six6*, *Jag2*, *Lin7c*, *Pdcd4*, *Csf1*, *Topors*, *Spg21*, *Akt1*, *Egfr*, *Sox10*, *C2*, *Scn5a*, *Arhgef4*, *Cdk2*
		protein localization (GO:0008104)	1.49E-03	3.77E-02	7	*Topors*, *Akt1*, *Sar1a*, *Egfr*, *Timm23*, *Tp53*, *Lin7c*
		cell division (GO:0051301)	2.04E-03	3.88E-02	3	*Ccnb2*, *Cdc42*, *Cdk2*
		cell cycle (GO:0007049)	3.75E-03	5.14E-02	7	*Pdcd4*, *Egfr*, *Cdk2*, *Cdkn2a*, *Ccnb2*, *Tp53*, *Jag2*
		nitrogen compound metabolic process (GO:0006807)	4.06E-03	5.14E-02	4	*Chst4*, *Akt1*, *Egfr*, *Chi3l1*
		cell proliferation (GO:0008283)	8.32E-03	8.99E-02	6	*Csf1*, *Topors*, *Egfr*, *Cdk2*, *Tp53*, *Jag2*
		death (GO:0016265)	9.46E-03	8.99E-02	6	*Akt1*, *Cdkn2a*, *Tp53*, *Jag2*, *Pdcd4*, *Topors*
	
	4	cell aging (GO:0007569)	4.38E-06	9.42E-04	3	*Pdcd4*, *Cdkn2a*, *Tp53*

^1 ^Only GO categories with False Discovery Rate (FDR) adjusted P-value < 0.1 and represented by three or more genes.

**Table 10:** Gene Ontology categories enriched among the genes associated with overall glioblastoma survival

Gene Ontology	Level	Term	P-value	FDR Adjusted P-value	Number of genes	Genes
Biological process	4	anatomical structure morphogenesis (GO:0009653)	5.39E-05	1.16E-02	9	*Nkx2-5*, *Csf1*, *Mapk3*, *Tgfa*, *Thbs4*, *Jag2*, *Igf2bp3*, *Myo7a*, *Hras*
	
	6	organ morphogenesis (GO:0009887)	3.83E-06	2.40E-03	7	*Nkx2-5*, *Csf1*, *Mapk3*, *Tgfa*, *Jag2*, *Myo7a*, *Hras*

^1 ^Only GO categories with False Discovery Rate (FDR) adjusted P-value < 0.1 and represented by three or more genes.

**Table 11:** Gene Ontology categories enriched among the genes associated with progression-free survival

Gene Ontology	Level	Term	P-value	FDR Adjusted P-value	Number of genes	Genes
Biological process	3	cell cycle (GO:0007049)	3.89E-06	2.96E-04	11	*Hras*, *Ppp1r15a*, *App*, *Calm2*, *Atf5*, *Pten*, *E2f3*, *Wee1*, *Tp53*, *Ccnd1*, *Nras*
		death (GO:0016265)	3.18E-04	1.21E-02	9^2^	*App*, *Raf1*, *Atf5*, *Pten*, *Fadd*, *Hspa1a*/*Hspa1b*, *Tp53*, *Ppp1r15a*
		response to biotic stimulus (GO:0009607)	4.11E-03	5.13E-02	6^2^	*Fadd*, *Hspa1a*/*Hspa1b*, *Clec2b*, *Ccnd1*, *Ifngr1*
		response to abiotic stimulus (GO:0009628)	8.17E-03	6.90E-02	6^2^	*Fadd*, *Hspa1a*/*Hspa1b*, *Clec2b*, *Ccnd1*, *Ifngr1*
	
	4	cell cycle process (GO:0022402)	4.26E-06	9.16E-04	10	*App*, *Atf5*, *Pten*, *E2f3*, *Wee1*, *Tp53*, *Ccnd1*, *Nras*, *Hras*, *Ppp1r15a*
	
	5	regulation of cell cycle (GO:0051726)	2.90E-07	1.22E-04	10	*Wee1*, *Tp53*, *Ccnd1*, *App*, *Nras*, *Hras*, *Ppp1r15a*, *Atf5*, *Pten*, *E2f3*
	
	6	regulation of progression through cell cycle (GO:0000074)	1.47E-07	9.24E-05	10	*Tp53*, *Ccnd1*, *Nras*, *Hras*, *Ppp1r15a*, *App*, *Atf5*, *Pten*, *E2f3*, *Wee1*
		cell death (GO:0008219)	2.13E-04	6.18E-02	9^2^	*Ppp1r15a*, *App*, *Raf1*, *Atf5*, *Pten*, *Fadd*, *Hspa1a*/*Hspa1b*, *Tp53*
	
	7	programmed cell death (GO:0012501)	1.19E-04	2.23E-02	9^2^	*Ppp1r15a*, *App*, *Raf1*, *Atf5*, *Pten*, *Fadd*, *Hspa1a*/*Hspa1b*, *Tp53*
	8	apoptosis (GO:0006915)	1.41E-04	5.21E-02	9^2^	*Ppp1r15a*, *App*, *Raf1*, *Atf5*, *Pten*, *Fadd*, *Hspa1a*/*Hspa1b*, *Tp53*

Molecular function	3	pattern binding (GO:0001871)	3.26E-04	3.55E-02	3	*Fstl1*, *App*, *Fgfr2*
		carbohydrate binding (GO:0030246)	1.26E-03	6.89E-02	4	*Fstl1*, *App*, *Fgfr2*, *Clec2b*
	
	4	polysaccharide binding (GO:0030247)	3.01E-04	8.70E-02	3	*Fstl1*, *App*, *Fgfr2*

^1 ^Only GO categories with False Discovery Rate (FDR) adjusted P-value < 0.1 and represented by three or more genes;

^2 ^Although *Hspa1a*/*Hspa1b *are represented by the same probe (202581_at), these isoforms are counted as two units.

### Cross-validation

The performance of the gene sets as reliable prognosticators of the three glioblastoma survival variables was evaluated. The generalization capability of the biomarker index was tested in individuals other than those used to develop a hazard index using a leave-one-individual-out discriminant analysis. Individuals were predicted to pertain to the high or low glioblastoma hazard groups for each event using the predictive biomarker index, and the prediction was compared to the observed classification based on the length of the period corresponding to each event. For both, lifetime and overall hazard, the number of observed high and low individuals was 100, and the number of predicted high and low individuals was 97 and 103, respectively. For the progression-free hazard, the number of observed high and low individuals was 87 and 88, respectively, and the number of predicted high and low individuals was 83 and 92, respectively.

Additional evaluation of the results was pursued by comparing the genes associated with the three glioblastoma hazards identified in this study and the target genes of microRNAs associated with the glioblastoma hazard reported by Delfino et al. [43]. One third of the sequences identified in this study are putative targets of microRNAs associated with glioblastoma. A hypergeometric test confirmed that the overlap between the genes uncovered in this study and the target genes was significant (P-value ≤ 0.005).

## Discussion

The data set analyzed offered a suitable representation of the general population of glioblastoma cases. The median overall survival was 13 months, and the probabilities of survival at 12, 24, 36, 48 and 60 months post-diagnosis were 0.59, 0.25, 0.15, 0.11 and 0.07 respectively, in this study. The median survival is similar to that reported by Krex et al. [14], and the 60 month survival probability is comparable to the 5-year survival rate of 0.13 estimated for grade IV brain cancer reported by the National Cancer Institute Surveillance Epidemiology and End Results [44]. The similarity between the survival rate in this study and that reported for primary glioblastoma suggests an insignificant fraction of secondary glioblastoma samples among the samples analyzed [20].

Comparing findings against a literature review confirmed that the Cox survival analysis of multiple gene expression profiles and clinical variables simultaneously was an effective tool to detect an integrated set of gene expression profiles exhibiting general and cohort-dependent associations with the three glioblastoma survival variables. The majority of the genes associated with lifetime, overall, and progression-free survival, in this study, have been previously reported to be associated with glioblastoma (35, 24, and 35 genes, respectively) or with another cancer (10, 19, and 15, respectively). In addition, the multi-factor analysis and data used in this study allowed the uncovering several novel associations between gene profiles and glioblastoma survival. Specifically, 16, 4, and 10 previously unreported genes were associated with lifetime, overall, and progression-free survival, respectively in the present work. The discussion of the findings from our study is divided into genes associated with multiple survival variables, genes associated with glioblastoma in a cohort-independent or cohort-dependent manner, and further investigation of complex associations.

*Pik3r1 *and *E2f3 *were associated with all three glioblastoma survival variables (Tables 2, 3, 5 and 7). The higher glioblastoma hazards associated with higher levels of *Pik3r1 *observed in this study are supported by previous work showing that over-expression of this gene plays a role in the activation of the PI3K/Akt pathway resulting in cell proliferation and tumor invasion [45]. Likewise, a link between *E2f3 *and glioblastoma has been reported [28,46]. Among the 15 genes associated with two glioblastoma events (Table 2), *Akr1c3*, *Csf1*, *Jag2*, *Plcg1*, *Rpl37a*, *Sod2*, and *Topors *were associated with lifetime and overall survival (Tables 3 and 5). *Jag2 *has been associated with adenomas [47], pancreatic [48] and breast cancer [49], *Rpl37a *with nasopharyngeal carcinoma cell lines [50], and the rest with glioblastoma [26,51-54]. The consistent findings across both glioblastoma survival events suggest that these genes may have specific roles in death. Likewise, the association between *Hras *and overall and progression-free survival (Tables 2, 5 and 7), is consistent with previous glioblastoma studies [55] and suggests that this gene may have a role in aggressive glioblastoma growth. *Fstl1*, *Mtap*, *Tp53*, *Camk2g *214322_at, and *Il13ra1 *probe 210904_s_at, were associated with lifetime and progression-free survival (Tables 2, 3 and 7) and these associations are supported by previous studies [20,24,25,28,56-58].

Most genes (lifetime survival, 55 out of 61 genes; overall survival, 45 out of 47 genes; and progression-free survival, 55 out of 60 genes) were associated with survival in a general or cohort-independent manner. The most extreme cohort-independent changes in lifetime survival were observed in *Syne1 *(HR = 0.17) and *Pdcd4 *(HR = 4.68), and the former profile has been found in lung [59], ovarian [60], colon, and breast cancers [61]; while, the second has been associated with glioma [62]. The most extreme cohort-independent changes in overall survival were observed in *Ighg1 *(HR = 4.33) and *Tgfa *(HR = 0.12), and the former trend has been found in cancer cell lines [63]; meanwhile the later is present in the KEGG glioma pathway [28]. Lastly, the genes that presented extreme hazard ratio values and general association with progression-free survival are *Pla2g7 *(HR = 0.11) and *Paics *(HR = 5.28). The *Pla2g7 *and *Paics *trends identified in this study are consistent with those reported for breast cancer in mice [64] and in non-glioma types of cancer [65,66], respectively.

Several genes (lifetime survival, 6 out of 61 genes; overall survival, 2 out of 47 genes; and progression-free survival, 5 out of 60 genes) were associated with glioblastoma survival in a cohort-dependent manner. These findings indicate that effective use of these genes in prognostic indices or in therapy development must consider the personal characteristics of the individual. Higher levels of *C2 *and *Prkcb *(probe 209685_s_at) were associated with a higher lifetime death hazard in males (HR = 1.93 and 5.22, respectively) than in females (HR = 1.30 and 1.31, respectively) and the profile of the latter gene has been observed in colon cancer cell lines [67]. The lifetime hazard estimate decreased with increased levels of *Sox10 *in Caucasian individuals (HR = 0.55) compared to non-Caucasian individuals, and this pattern is concordant with broad distribution of *Sox10 *in high grade gliomas [68]. Increases in the level of *Chi3l1 *were associated with significant increases in lifetime hazard estimates across all therapies with the highest hazard ratio observed in individuals receiving no therapy (None, HR = 2.42). This trend is consistent with reports that *Chi3l1*/*Ykl-40 *was highly overexpressed in glioblastoma relative to nonneoplastic brain [69] and that *Ykl-40 *is associated with poorer response to radiation and shorter lifetime survival in glioblastoma [70]. Males (HR = 0.36) and individuals receiving no therapy (HR = 0.38) have the lowest hazard ratio per increase in *Prkcb *(probe 207957_s_at). These trends are consistent with those reported for other cancer types [67] and with observations of protein kinase C activation in gamma-irradiated proliferating and confluent human lung fibroblast cells [71].

The cohort-dependent associations between overall survival and both *Polr2d *and *Igf2bp3 *have been observed in colorectal cancer [72] and glioblastoma [73], respectively. Three genes (*Rab31*, *Rps20 *and *Apool*) exhibited a cohort-dependent association with overall survival that is consistent with previously reported trends [74-76]. Lastly, the gender-dependent association between *Gdf10 *and progression-free survival is in agreement with reports of copy number loss of *Gdf10 *in mesothelioma [77].

Further analyses of the association between individual genes (with or without clinical variables) and hazards were undertaken when the trend estimated from the multi-gene index was opposite to that previously reported. Nine genes and survival events were re-analyzed individually and compared to previous reports including: *E2f3 *and all three survival variables [28,46], *Egfr *and lifetime survival [78], *Cfs1 *and lifetime survival, *Mdm2 *with overall hazard [79], *Fstl1 *and lifetime and progression-free survival [25], *Mtap *and progression-free hazard [57], *Pdcd4 *and lifetime survival [62], *Tgfa *and overall survival [80], and race-dependent *Vav3 *and overall survival [81]. In the first six cases, the consideration of the gene alone as predictor of glioblastoma survival as standard in previous reports resulted in non-significant associations, in this study. These results indicate that the accurate identification of biomarkers and precise characterization of the trend requires the study of the genes in concert with other genes in a systems biology framework, such as the approach implemented, in this study. Re-analysis of *Pdcd4 *and *Vav3 *confirmed the significant trend detected in the multi-gene analysis, suggesting that further studies are needed to precisely characterize the trend.

The LOOCV confirmed the adequacy of the set of genes and clinical variables identified to predict the glioblastoma hazards. The minor differences between the observed and predicted numbers in each group may be due to the discretization of the survival length into high and low groups required by the discriminant analysis; whereas, the Cox survival analysis models continuous time to the glioblastoma event. The significant number of genes prognostic of glioblastoma survival identified in this study that are also targets of microRNAs associated with glioblastoma [43] further confirms our results.

In addition to literature review and LOOCV, the gene-survival associations detected in this study were confirmed using the information from the REMBRANDT database. The associations between survival and the 10 gene probes with the most extreme hazard ratio estimate for each of the three survival variables studied that did not interact with cohort variables (Tables 3, 5, 7) were investigated in REMBRANDT. The query was performed using the Kaplan-Meier survival plot for Gene Expression Data. Of these, eight genes had the same significant trend observed in our study (*Syne1*, *Gigyf2*/*Tnrc15*, *Scn5a*, *Hoxa10*, *Pdcd4*, *Tgfa*, *Pla2g7 *and *Agpat1*), two did not have information on the REMBRANDT database (*Ighg1 *and *Hnrnpd*), *Fstl1 *had an opposite trend than the one observed in our study and in previous independent studies (Table 3) and most of the remaining genes, although non-significant, had the same trend observed in our analysis. The latter results are consistent with the simpler analytical approach based on Kaplan-Meier curves available in REMBRANDT, when compared to the more flexible Cox survival analysis used in our study. The Kaplan-Meier approach relies on non-parametric rank-based test to compare the survival between individuals with high and low gene expression. These groups are obtained by setting up an arbitrary expression threshold. Non-parametric rank-based approaches tend to have lower power to detect significant variation than semi- and parametric approaches such as the Cox survival analysis. In addition, the Kaplan-Meier analysis only allows the consideration of one explanatory variable at a time, and this variable has to be discrete (thus, the reason for comparing high and low expression groups in REMBRANDT). This approach does not allow considering multiple continuous covariates (i.e. gene expression) and factors (e.g. race, gender, therapy and progression) or interactions simultaneously. The Cox-survival analysis implemented in our study allows the simultaneous consideration of multiple factors (such as possible population stratification due to race), covariates (e.g. other gene expression profiles) and interactions, and it does not require the discretization of the gene expression values that could result in potential loss of information. Thus, the Cox approach used in our study is able to capture the association between continuous gene expression values and survival conditional on all other model terms and is able to detect associations that are likely not to reach statistical significance using the Kaplan-Meier comparison of survival between high and low gene expression groups.

Among the GO categories, 19 biological processes and three molecular functions were over-represented (FDR adjusted P-value < 0.1, ≥ 3 genes per category) in the genes associated with the three glioblastoma events studied (Tables 9, 10 and 11). Two biological processes, cell cycle (GO:0007049) and death (GO:0016265), were over-represented in the lifetime and progression-free survival (Tables 9 and 11), and several biological processes have been previously associated with glioblastoma [17,62,68,70,79,82-86]. These processes included: aging, morphogenesis, cell cycle and proliferation, and death for lifetime survival; morphogenesis for overall survival; and cell cycle, death and recognition, death, response to biotic and abiotic stimuli, programmed cell death, and apoptosis for progression-free survival.

The study of complementary glioblastoma survival variables allowed to confirm that the gene profiles associated with lifetime survival resulting in the enriched functional category of aging are clearly associated with cancer initiation and progression and are not a simply reflection of the natural aging process. Two results confirm that the biomarkers are not mere confounding with aging. First, the genes in the GO terms "aging (GO:0007568)" and "cell aging (GO:0007569)", *Pdcd4*, *Cdkn2a*, and *Tp53*, have all been associated with GBM in previous independent studies (Table 3). In addition, *Tp53 *was associated with progression-free survival (Table 7). Second, other functional terms enriched among the genes associated with lifetime glioblastoma survival were also identified on the other glioblastoma survival variables studied. The biological processes of cell death and cell cycle were enriched both for lifetime and progression-free survival.

The biological processes, molecular functions and gene networks particular to a glioblastoma survival event offered insights into the processes particular to the initiation and progression of this cancer. For instance, eight biological processes associated with lifetime survival were level 3, and one was level 4, indicating that the differentially expressed genes associated with lifetime survival participate on broad or general biological mechanisms. The interconnection between the genes pertaining to aging further confirms the significance of this gene network on lifetime survival (Figure 4). Although only two biological processes were associated with overall survival, these processes correspond to levels 4 and 6. This result indicates that the genes associated with overall survival correspond to more specific mechanisms. Moreover, both biological processes are related to generation and organization of anatomical structures, such as organs, and this finding may be associated to the dispersion and development of malignant cells after diagnosis and resection. The close relationship between biomarker genes in this network supports this finding (Figure 5). Albeit the study of progression-free survival encompassed a shorter period than lifetime and overall survival, the functional analysis showed several biological processes and molecular functions over-represented among the genes associated with this survival. Four of the biological processes are from level 6 to 8, indicating that specific gene networks and roles are associated with progression-free survival. The biological processes associated with progression-free survival include regulation of progression through cell cycle, programmed cell death, and apoptosis. Extensive relationships between the biomarker genes in the cell cycle were identified further, supporting the major role of this network on glioblastoma progression (Figure 6). In addition, three molecular functions were enriched among the genes associated with progression-free survival. Therefore, many biological and molecular events occur in the period between the diagnosis of malignancy and progression or recurrence, probably due to response to numerous treatments, surgery, and cancer progression. Two genes were highly represented across the categories (Tables 9 to 11). *Tp53 *has an important role as a tumor repressor [83], and *App *is highly expressed in individuals with short-term glioblastoma survival [24].

## Conclusions

An innovative approach to identify simultaneously multiple biomarkers of lifetime, overall and progression-free glioblastoma survival in a systems biology framework was presented. Furthermore, the inclusion of clinical information allowed the uncovering of general and individualized associations between gene expression profiles and three complementary survival metrics. This study demonstrated the pre-eminence of developing multi-gene prognostic indices of glioblastoma survival through the integration of variable selection and survival models relative to the simple-yet- simplistic single-gene analysis. Known biomarker gene profiles were confirmed, and new general and clinical-dependent gene profiles were uncovered. The present study looked at glioblastoma in general and complements work on the identification of genes associated with specific glioblastoma types [42,87,88]. Empirically confirmed findings will be the basis for improved prognostic tools and individualized treatments that improve the survival and quality of life of individuals suffering glioblastoma multiforme.

## Competing interests

The authors declare that they have no competing interests.

## Authors' contributions

NVLS compiled the data; performed the normalization, survival, and functional analyses; contributed to the interpretation of results; and drafted the manuscript. KRD participated in the analyses. BRS participated in the analyses and contributed in drafting the manuscript. JEB obtained funding for the study and helped in the drafting of the manuscript. SRZ obtained funding for the study and permission to use the data; participated in its conception, coordination, and interpretation of results; and helped to draft the manuscript. All authors have read and approved the final version of this manuscript.

## Pre-publication history

The pre-publication history for this paper can be accessed here:

http://www.biomedcentral.com/1755-8794/4/49/prepub

## Supplementary Material

Additional file 1 **List of genes associated in glioblastoma from the literature** Table containing the list of 174 genes previously reported in the literature. Click here for file
