# Supplementary material for: Cell cycle and aging, morphogenesis, and response to stimuli genes are individualized biomarkers of glioblastoma progression and survival
Source: BMC Med Genomics. 2011 Jun 7;4:49. doi: 10.1186/1755-8794-4-49 (PMC3127972; doi:10.1186/1755-8794-4-49)
Supplement: Additional file 1 — List of genes associated in glioblastoma from the literature Table containing the list of 174 genes previously reported in the literature. [file 1755-8794-4-49-S1.DOCX]

| Reference | Number of genes | Genes |
| --- | --- | --- |
| Bredel et al. (2009) | 42^1,2,3,4^ | *Abcc4*, *Actn4*, *Akr1c3*, *Anxa7*, *Bax*, *Casp3*, *Cdc42*, *Cdk6*, *Csf1*, *Cul1*, *Cycs*, *Egfr*, *Eif6*, *Ep300*, *Ewsr1*, *Fgfr2*, *Gbas*, *Igf1r*, *Mdm2*, *Mfn2*, *Mgat3*, *Mtap*, *Mxi1*, *Mybl2*, *Myc*, *Pak4*, *Pcna*, *Pdcd4*, *Plcg1*, *Pold2*, *Ppp1r15a*, *Pten*, *Pvr*, *Raf1*, *Rala*, *Rangap1*, *Sirpa*, *Smarcb1*, *Timp3*, *Topors*, *Wdr11* and *Yme1l1* |
| The Cancer Genome Atlas Research Network (TCGA, 2008) | 21^1,3,5,6^ | *Akt3*, *Cdkn2a*, *Cdkn2b*, *Egfr*, *Erbb2*, *Fgfr2*, *Irs2*, *Met*, *Mlh1*, *Msh2*, *Msh6*, *Nf1*, *Park2*, *Pdgfra*, *Pik3ca*, *Pik3r1*, *Pms2*, *Pten*, *Ptprd*, *Rb1* and *Tp53* |
| KEGG Glioma Pathway | 51^6^ | *Akt1*, *Akt2*, *Akt3*, *Braf*, *Calm1*, *Calm2*, *Calm3*, *Calml3*, *Calml5*, *Camk2a*, *Camk2b*, *Camk2g*, *Ccnd1*, *Cdk4*, *Cdkn1a*, *Cdkn2a*, *E2f1*, *E2f2*, *E2f3*, *Egf*, *Grb2*, *Hras*, *Igf1*, *Kras*, *Map2k1*, *Map2k2*, *Mapk3*, *Mtor*, *Nras*, *Pdgfa*, *Pdgfb*, *Pdgrfa*, *Pdgrfb*, *Pik3ca*, *Pik3cb*, *Pik3cd*, *Pik3cg*, *Pik3r1*, *Pik3r2*, *Pik3r3*, *Pik3r5*, *Prkca*, *Prkcb*, *Prkcg*, *Rb1*, *Shc1*, *Shc2*, *Shc3*, *Sos1*, *Sos2* and *Tgfa* |
| Marko et al. (2008) | 38^2,3,5^ | *Actr2*, *Adam2*, *Ank1*, *App*, *Araf*, *Bmx*, *Capn1*, *Cd34*, *Cpvl*, *Egfr*, *Fscn1*, *Gbas*, *Grm8*, *Hspa1b*, *Idh1*, *Ighg1*, *Il13ra1*, *Il17*, *Il22*, *Itga6*, *Jag2*, *Kcnq2*, *Kifc3*, *Lrp10*, *Mmp14*, *Ndrg1*, *Nos2*, *Padi3*, *Pcdha6*, *Pfdn1*, *Rpl10*, *Rpl41*, *Serping1*, *Shox2*, *Sox4*, *Tp53*, *Ucp3* and *Wnt1* |
| Reddy et al. (2008) | 7^5^ | *Aebp1*, *Chi3l1*, *Fstl1*, *Gadd45a*, *Rhoc*, *Sod2* and *Tp53* |
| Zhang et al. (2007) | 23^4^ | *Ccna2*, *Ccnb1*, *Ccnb2*, *Cdc45l*, *Cdc6*, *Cdc7*, *Cdk2*, *Cks1b*, *Dnmt1*, *Ggh*, *Ifngr1*, *Mcm6*, *Pcna*, *Pttg1*, *Rac2*, *Rpa2*, *Rpa3*, *Rrm1*, *Rrm2*, *Tubg1*, *Tyms*, *Ung* and *Wee1* |

^1^Genes *Fgfr2* and *Pten* are represented by Bredel et al. (2009) and TCGA (2008);

^2^Gene *Gbas* is represented by Bredel et al. (2009) and Marko et al. (2008);

^3^Gene *Egfr* is represented by Bredel et al. (2009), TCGA (2008) and Marko et al. (2008);

^4^Gene *Pcna* is represented by Bredel et al. (2009) and Zhang et al. (2007);

^5^Gene *TP53* is represented by TCGA (2008), Marko et al. (2008) and Reddy et al. (2008);

^6^Genes *Akt3* and *Cdkn2a* are represented by the KEGG Glioma Pathway and TCGA (2008).
